# Demographic Characteristics, Experiences, and Beliefs Associated with Hand Hygiene Among Adults During the COVID-19 Pandemic — United States, June 24–30, 2020

**DOI:** 10.15585/mmwr.mm6941a3

**Published:** 2020-10-16

**Authors:** Mark É. Czeisler, Amanda G. Garcia-Williams, Noelle-Angelique Molinari, Radhika Gharpure, Yiman Li, Catherine E. Barrett, Rebecca Robbins, Elise R. Facer-Childs, Laura K. Barger, Charles A. Czeisler, Shantha M.W. Rajaratnam, Mark E. Howard

**Affiliations:** ^1^Turner Institute for Brain and Mental Health, Monash University, Melbourne, Australia; ^2^Austin Health, Melbourne, Australia; ^3^Brigham and Women’s Hospital, Boston, Massachusetts; ^4^CDC COVID-19 Response Team; ^5^Division of Foodborne, Waterborne and Environmental Diseases, National Center for Emerging and Zoonotic Infectious Diseases, CDC; ^6^Harvard Medical School, Boston, Massachusetts; ^7^University of Melbourne, Melbourne, Australia.

Frequent hand hygiene, including handwashing with soap and water or using a hand sanitizer containing ≥60% alcohol when soap and water are not readily available, is one of several critical prevention measures recommended to reduce the spread of SARS-CoV-2, the virus that causes coronavirus disease 2019 (COVID-19).* Previous studies identified demographic factors associated with handwashing among U.S. adults during the COVID-19 pandemic (*1*,*2*); however, demographic factors associated with hand sanitizing and experiences and beliefs associated with hand hygiene have not been well characterized. To evaluate these factors, an Internet-based survey was conducted among U.S. adults aged ≥18 years during June 24–30, 2020. Overall, 85.2% of respondents reported always or often engaging in hand hygiene following contact with high-touch public surfaces such as shopping carts, gas pumps, and automatic teller machines (ATMs).^†^ Respondents who were male (versus female) and of younger age reported lower handwashing and hand sanitizing rates, as did respondents who reported lower concern about their own infection with SARS-CoV-2^§^ and respondents without personal experience with COVID-19. Focused health promotion efforts to increase hand hygiene adherence should include increasing visibility and accessibility of handwashing and hand sanitizing materials in public settings, along with targeted communication to males and younger adults with focused messages that address COVID-19 risk perception.

During June 24–30, among 9,896 eligible U.S. adults,^¶^ 5,412 (54.7%) completed Internet-based surveys administered by Qualtrics, LLC, as part of The COVID-19 Outbreak Public Evaluation (COPE) Initiative.** The Monash University Human Research Ethics Committee of Monash University (Melbourne, Australia) reviewed and approved the study protocol on human subjects research. This activity was also reviewed by CDC and was conducted consistent with applicable federal law and CDC policy.^††^ Respondents were informed of study purposes and provided electronic consent before commencement, and investigators received anonymized responses. The 5,412 participants who completed surveys during June included 3,683 (68.1%) first-time respondents and 1,729 (31.9%) respondents who were recontacted after having been recruited to participate in The COPE Initiative during April 2–8, 2020.^§§^ Complete data for explanatory variables included in the analysis were obtained from 5,000 (92.4%) respondents. Among these respondents, 4,817 (96.3%) reported having been in public during the previous week and were included in this analysis (3,243 [67.3%] first-time respondents and 1,574 [32.7%] recontacted respondents). Quota sampling and survey weighting were employed to improve sample representativeness of the adult U.S. population by gender, age, and race/ethnicity. Hand hygiene frequency was assessed on a five-item Likert scale from “Never” to “Always” using the following questions: “In the last week, how frequently did you use hand sanitizer after touching high-touch surfaces in public?” and “In the last week, how frequently did you wash your hands with soap and water after touching high-touch surfaces in public?” Bivariate chi-squared analyses identified covariates associated with frequency of hand hygiene.

With handwashing and hand sanitizing frequency as dependent variables for separate models, adjusted odds ratios (aORs) and 95% confidence intervals (CIs) for hand hygiene frequency were estimated using weighted ordered logistic regressions with the following explanatory variables: gender, age, race/ethnicity, 2019 household income, U.S. Census region,^¶¶^ rural/urban residence,*** whether respondents knew someone who had positive test results for SARS-CoV-2 or who was hospitalized for or died from COVID-19, and concern for personal risk for infection with SARS-CoV-2 (from “Not at all” to “Extremely”). Statistical analyses were conducted in R (version 4.0.2; The R Foundation) with the R survey package (version 3.29).

Among 4,817 U.S. adults, 85.2% reported frequent (always or often) use of at least one form of hand hygiene after contact with high-touch public surfaces, including handwashing (78.5%) and hand sanitizing (70.7%) (Table). Frequent handwashing and hand sanitizing were least prevalent among adults aged 18–24 years (64.6% and 59.8%, respectively, with 72.4% reporting at least one form of hand hygiene); frequency increased with age and was highest among persons aged ≥65 years (83.3% and 73.3%, respectively, with 89.4% reporting at least one form of hand hygiene). Frequent hand sanitizing was more prevalent among respondents with a 2019 household income ≥$100,000 (72.6%) compared with those with a household income <$25,000 (62.5%). Regarding concern for personal risk for SARS-CoV-2 infection, frequent handwashing and hand sanitizing were least prevalent among those not at all concerned (68.0% and 54.0%, respectively, with 72.1% reporting at least one form of hand hygiene); prevalence increased with level of concern and was most prevalent among those extremely concerned (89.5% and 83.1%, respectively, with 93.7% reporting at least one form of hand hygiene).

**TABLE:** Prevalence of frequent hand hygiene* after contact with high-touch public surfaces among adults, by select respondent characteristics — United States, June 24–30, 2020

Characteristic	All respondents	Often or always wash hands		Often or always use hand sanitizer	
	Weighted no. (%)^†^	Weighted no. (%)^†^	P-value^§^	Weighted no. (%)^†^	P-value^§^
**Overall**	4,817 (100)	3,781 (78.5)	—	3,407 (70.7)	—
**Demographic characteristic**					
**Sex**					
Female	2,448 (50.8)	1,971 (80.5)	<0.001	1,800 (73.5)	<0.001
Male	2,369 (49.2)	1,810 (76.4)		1,608 (67.9)	
**Age group, yrs**					
18–24	629 (13.1)	406 (64.6)	<0.001	376 (59.8)	<0.001
25–44	1,685 (35.0)	1,295 (76.8)		1,210 (71.8)	
45–64	1,672 (34.7)	1,388 (83.0)		1,212 (72.5)	
≥65	830 (17.2)	692 (83.3)		609 (73.3)	
**Race/Ethnicity**					
White, non-Hispanic	3,068 (63.7)	2,461 (80.2)	<0.001	2,208 (72.0)	<0.001
Black, non-Hispanic	587 (12.2)	427 (72.7)		385 (65.6)	
Asian, non-Hispanic	230 (4.8)	198 (86.2)		182 (79.0)	
Other or multiple race or races, non-Hispanic^¶^	145 (3.0)	104 (71.9)		95 (65.9)	
Hispanic, any race or races	787 (16.3)	590 (75.0)		537 (68.2)	
**2019 household income, USD**					
<$25,000	639 (13.3)	471 (73.6)	<0.001	400 (62.5)	<0.001
$25,000–$49,999	992 (20.6)	765 (77.1)		707 (71.3)	
$50,000–$99,999	1,670 (34.7)	1,343 (80.4)		1,200 (71.9)	
≥$100,000	1,515 (31.5)	1,202 (79.4)		1,100 (72.6)	
U.S. Census region**					
Northeast	1,073 (22.3)	862 (80.3)	0.941	747 (69.6)	0.044
Midwest	913 (19.0)	710 (77.7)		646 (70.7)	
South	1,674 (34.7)	1,300 (77.7)		1,217 (72.7)	
West	1,157 (24.0)	909 (78.6)		797 (68.9)	
**Rural/Urban residence^††^**					
Rural	544 (11.3)	423 (77.8)	0.003	396 (72.7)	0.211
Urban	4,273 (88.7)	3,358 (78.6)		3,012 (70.5)	
**COVID-19 experiences and beliefs**					
**Knew someone who had test results positive for SARS-CoV-2**					
Yes	970 (20.1)	837 (86.4)	<0.001	771 (79.5)	<0.001
No	3,847 (79.9)	2,944 (76.5)		2,636 (68.5)	
**Knew someone who was hospitalized for severe illness or died from COVID-19**					
Yes	624 (12.9)	518 (83.0)	0.002	495 (79.4)	<0.001
No	4,193 (87.1)	3,263 (77.8)		2,912 (69.4)	
**Level of concern of own risk of SARS-CoV-2 infection^§§^**					
Not at all	576 (12.0)	392 (68.0)	<0.001	311 (54.0)	<0.001
Slightly	1,093 (22.7)	810 (74.1)		727 (66.5)	
Moderately	1,411 (29.3)	1,086 (77.0)		966 (68.5)	
Very	783 (16.2)	639 (81.6)		610 (77.9)	
Extremely	954 (19.8)	854 (89.5)		793 (83.1)	

**Abbreviations:** COVID-19 = coronavirus disease 2019; USD = U.S. dollars.

* Frequency of hand hygiene was assessed on a 5-point Likert scale from “Never” to “Always” using the following questions: “In the last week, how frequently did you use hand sanitizer after touching high-touch surfaces in public” and “In the last week, how frequently did you wash hands with soap and water after touching high-touch surfaces in public.” For this table, answers of “Often” or “Always” were considered frequent.

^†^ Quota sampling and survey weighting were employed to improve representativeness of the cross-sectional June cohort of the United States population by gender, age, and race/ethnicity according to the 2010 U.S. Census.

^§^ Bivariate chi-squared test was used to test for differences in observed and expected frequencies among groups by characteristic for each type of hand hygiene on the full 5-item Likert scale from “Never” to “Always.” Statistical significance for bivariate analyses was evaluated as p<0.05.

^¶^ The non-Hispanic, other race or multiple races category includes respondents who identified as not Hispanic and as more than one race or as American Indian or Alaska Native, Native Hawaiian or Pacific Islander, or Other.

** Region classification was determined using the U.S. Census Bureau’s Census Regions and Divisions of the United States. https://www2.census.gov/geo/pdfs/maps-data/maps/reference/us_regdiv.pdf.

^††^ Rural/urban residence was classified as urban or rural based on self-reported ZIP codes according to the Federal Office of Rural Health Policy definition of rurality. https://www.hrsa.gov/rural-health/about-us/definition/datafiles.html.

^§§^ For this question, respondents were asked to rate on a scale from “Not at all” to “Extremely” the extent to which they were concerned about the following statement regarding COVID-19 and infection control measures: “My own risk of infection with COVID-19.”

The aORs and 95% CIs reflect significant differences in odds of more frequent handwashing associated with gender, age, race/ethnicity, whether the respondent knew someone who had received a positive SARS-CoV-2 test result, and concern for personal risk for SARS-CoV-2 infection (Figure 1). Odds of more frequent handwashing were lower for males than for females (aOR = 0.65; 95% CI = 0.57–0.74) and higher among older than among younger respondents (e.g., aOR = 2.36; 95% CI = 1.85–3.01 for persons aged 45–64 years compared with those aged 18–24 years). Odds of more frequent handwashing were 66% higher among non-Hispanic Asian respondents than among non-Hispanic White (White) respondents (aOR = 1.66; 95% CI = 1.34–2.06) and were 30% higher among those who knew someone who received a positive SARS-CoV-2 test result than among those who did not (aOR = 1.30; 95% CI = 1.10–1.53). Compared with those who were not at all concerned about SARS-CoV-2 infection, those who were moderately, very, and extremely concerned had 35% (aOR = 1.35; 95% CI = 1.07–1.72), 77% (aOR = 1.77; 95% CI = 1.36–2.31), and 209% higher odds (aOR = 3.09; 95% CI = 2.38–4.01), respectively, of more frequent handwashing.

**FIGURE 1:**
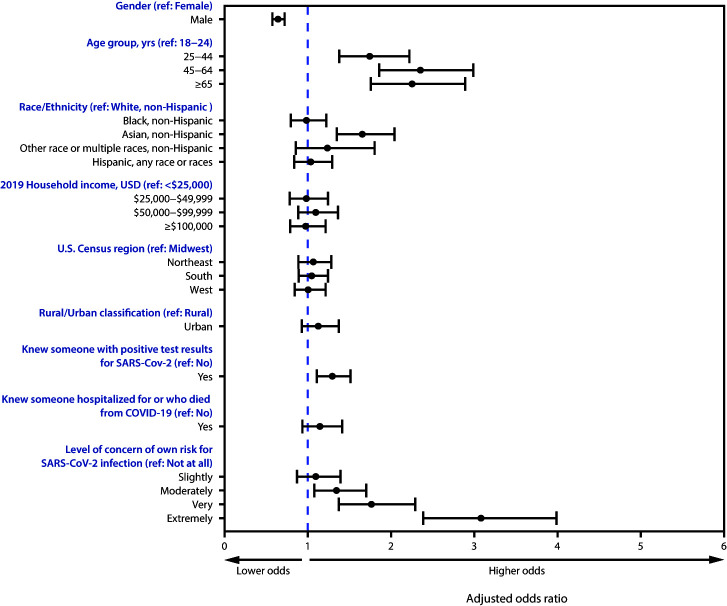
Adjusted odds ratios*^,†^ for washing hands after contact with high-touch public surfaces,^§^ by select respondent characteristics^¶^^,^**^,^^††^^,^^§§^ — United States, June 24–30, 2020 **Abbreviations:** COVID-19 = coronavirus disease 2019; ref = referent; USD = U.S. dollars. * Adjusted odds ratios were estimated using an ordered logit model of handwashing on the variables listed in the column with a proportional odds assumption. ^†^ 95% confidence intervals indicated with error bars. ^§^ Frequency of handwashing was assessed on a 5-point Likert scale from “Never” to “Always” using the following question: “In the last week, how frequently did you wash your hands with soap and water after touching high-touch surfaces in public.” ^¶^ The non-Hispanic, other race, or multiple races category includes respondents who identified as not Hispanic and as more than one race or as American Indian or Alaska Native, Native Hawaiian or Pacific Islander, or Other. **Region classification was determined using the U.S. Census Bureau’s Census Regions and Divisions of the United States. https://www2.census.gov/geo/pdfs/maps-data/maps/reference/us_regdiv.pdf. ^††^ Rural/urban residence was classified as urban or rural based on self-reported ZIP codes according to the Federal Office of Rural Health Policy definition of rurality. https://www.hrsa.gov/rural-health/about-us/definition/datafiles.html. ^§§^ For this question, respondents were asked to rate on a scale from “Not at all” to “Extremely” the extent to which they were concerned about the following statement regarding COVID-19 and infection control measures: “My own risk of infection with COVID-19.”

Adjusted odds of more frequent hand sanitizing were similar to those observed for more frequent handwashing (Figure 2), with the following exceptions: those with higher 2019 household income ($25,000–$49,999) had 30% higher odds of more frequent hand sanitizing (aOR = 1.30, 95% CI = 1.04–1.64) than did those with household income <$25,000, and those who knew someone hospitalized for or who died from COVID-19 had 28% higher odds of more frequent hand sanitizing (aOR = 1.28; 95% CI = 1.04–1.59) than did those who did not know someone who had been hospitalized or died from COVID-19.

**FIGURE 2:**
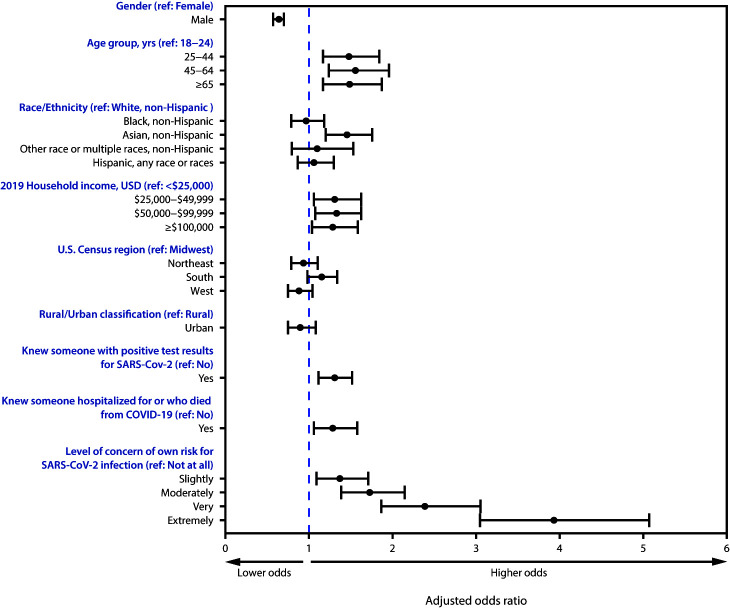
Adjusted odds ratios*^,†^ for use of hand sanitizer after contact with high-touch public surfaces,^§^ by select respondent characteristics^¶^^,^**^,^^††^^,^^§§^ — United States, June 24–30, 2020 **Abbreviations:** COVID-19 = coronavirus disease 2019; ref = referent; USD = U.S. dollars. * Adjusted odds ratios were estimated using an ordered logit model of using hand sanitizer on the variables listed in the column with a proportional odds assumption. ^†^ 95% confidence intervals indicated with error bars. ^§^ Frequency of hand sanitizing was assessed on a 5-point Likert scale from “Never” to “Always” using the following question: “In the last week, how frequently did you use hand sanitizer after touching high-touch surfaces in public after touching high-touch surfaces in public.” ^¶^ The non-Hispanic, other race, or multiple races category includes respondents who identified as not Hispanic and as more than one race or as American Indian or Alaska Native, Native Hawaiian or Pacific Islander, or Other. ** Region classification was determined using the U.S. Census Bureau’s Census Regions and Divisions of the United States. https://www2.census.gov/geo/pdfs/maps-data/maps/reference/us_regdiv.pdf. ^††^ Rural/urban residence was classified as urban or rural based on self-reported ZIP codes according to the Federal Office of Rural Health Policy definition of rurality. https://www.hrsa.gov/rural-health/about-us/definition/datafiles.html. ^§§^ For this question, respondents were asked to rate on a scale from “Not at all” to “Extremely” the extent to which they were concerned about the following statement regarding COVID-19 and infection control measures: “My own risk of infection with COVID-19.”

## Discussion

Approximately 85% of 4,817 U.S. adults frequently engaged in either handwashing or using hand sanitizer after contact with high-touch public surfaces, including only 72.4% of those aged 18–24 years. These findings highlight the need for continued health communication and outreach promoting hand hygiene. Respondents who were male and of younger age reported less frequent handwashing and hand sanitizing. These findings are consistent with those from previous pandemics (*3*) and earlier in the COVID-19 pandemic (*1*), when males and younger adults engaged in less frequent handwashing than did females and older adults (*2*,*3*). During the COVID-19 pandemic, one study found that Hispanic adults reported more frequent handwashing than did White adults (*1*); however, the current study did not find a difference in handwashing between Hispanic and White adults after adjusting for concern for SARS-CoV-2 infection.

Respondents with lower income reported less frequent hand sanitizing. This could reflect lack of access to hand sanitizer; higher income and access to handwashing infrastructure have been previously found to be associated with adherence to hand hygiene (*4*). Difficulty obtaining hand sanitizer has been documented during the COVID-19 pandemic (*5*), and purchasing hand sanitizer might be prohibitive for persons with low income, particularly given recent reported increases in cost.^†††^ Strategies to increase hand sanitizing among lower-income populations could apply innovative approaches with regard to the location of signage and contactless dispensers (e.g., the center of a lobby or market or next to or built into gas filling stations) to make hand sanitizer and handwashing materials visible and readily available in public settings and address disparities in access.

Increased concern for personal risk for SARS-CoV-2 infection and personal experience with COVID-19 were both positively associated with handwashing and hand sanitizing. During previous respiratory pandemics, general concern, perceived susceptibility, and perceived severity of illness were found to be positively associated with engagement in hygiene-related prevention behaviors (*3*). During this pandemic, higher perceived risk has been associated with increased handwashing (*6*). In addition to hand hygiene, risk perceptions have been associated with engaging in other protective behaviors such as physical distancing,^§§§^ avoiding handshakes and crowds (*7*), and wearing cloth face masks (*8*). Perceived risk for COVID-19 in the United States, when assessed during March–April 2020, was moderately high (*6*); however, some evidence indicates U.S. adults underestimate their risk of becoming ill with COVID-19 (*7*). Differences in risk perceptions might partially explain why men and younger adults reported less frequent practicing of hand hygiene compared with women and older adults. Although differences in risk perceptions by gender and age were not assessed in this study, research conducted during the COVID-19 pandemic has found that younger persons (*7*,*9*) and men (*6*) had lower COVID-19 risk perceptions compared with older adults and women. For both populations, efforts are needed to further characterize COVID-19 risk perceptions and their relationships to hand hygiene, and to identify how health communication efforts can address risk perceptions in promotion of preventive behaviors. This is particularly important given that only 72.1% of those who were not at all concerned about their risk for SARS-CoV-2 infection frequently engaged in either handwashing or using hand sanitizer after contact with high-touch public surfaces, compared with 93.7% of those who were extremely concerned.

The findings in this report are subject to at least five limitations. First, self-reported data are subject to recall, response, and social desirability biases, and self-reported hand hygiene behavior might be overreported. Survey weighting might not have eliminated nonresponse bias. Second, estimation assumed proportional odds (i.e., that odds are constant across response levels), an assumption that is often violated (*10*); weighted ordered logistic regressions were used for ease of interpretation given that the estimates did not differ substantially from models that did not assume proportional odds. Third, although quota sampling methods and survey weighting were employed to improve sample representativeness of 2010 U.S. Census adult population estimates for age, gender, and race/ethnicity, the Internet-based survey sample might not be fully representative of the 2020 U.S. population for income, educational attainment, and access to technology. Fourth, hand hygiene was self-reported by respondents after contact with high-touch public surfaces; future studies could evaluate hand hygiene within households, workplaces, and other environments. Similarly, although respondents included in this analysis had been in public during the preceding week, adherence to hand hygiene did not account for the number of times respondents contacted high-touch public surfaces, or the number of hand hygiene methods used following contact with such surfaces. Finally, respondents were not asked whether they had access to soap and water or hand sanitizer, which could influence hand hygiene behaviors.

Hand hygiene is part of a multicomponent public health approach, which also includes wearing face masks and maintaining a physical distance of ≥6 feet from others, among additional prevention measures, to prevent and control COVID-19 in community settings. Public health promotional outreach about hand hygiene is needed, given that these findings indicate that hand hygiene adherence could be improved, especially among certain groups. Hand-hygiene–related health promotion strategies should be tailored toward men and young adults. To motivate hand hygiene behavior, health promotion messaging could focus on addressing risk perceptions of COVID-19, which might have shared benefits to promote engagement in additional COVID-19 prevention measures. Finally, increasing visibility and accessibility of handwashing and hand sanitizing signage and materials in public settings could encourage and facilitate hand hygiene to prevent the spread of COVID-19.

**Summary**

**What is already known about this topic?**

Hand hygiene, including handwashing with soap and water and using hand sanitizer containing ≥60% alcohol, is one measure recommended to prevent COVID-19 and other infectious diseases.

**What is added by this report?**

In an Internet-based survey, approximately 85% of 4,817 U.S. adults reported frequent hand hygiene after contact with public surfaces. Males, young adults, respondents with lower concern about risk for SARS-CoV-2 infection, and respondents without personal COVID-19 experience reported less frequent hand hygiene.

**What are the implications for public health practice?**

COVID-19 messages should continue promoting hand hygiene, particularly among men and young adults. Messages addressing COVID-19 risk perceptions and making handwashing accessible and hand sanitizer available by facilities in public settings should be considered to encourage and facilitate hand hygiene.

## Footnotes

* https://www.cdc.gov/coronavirus/2019-ncov/prevent-getting-sick/prevention.html.

† Respondents were provided with the following examples as high-touch public surfaces: shopping carts, gas pumps, and ATMs.

§ For this question, respondents were asked to rate on a scale from “Not at all” to “Extremely” the extent to which they were concerned about the following statement regarding COVID-19 and infection control measures: “My own risk of infection with COVID-19.”

¶ Eligibility to complete a survey during June 24–30, 2020, was determined following electronic contact of potential participants with criteria of age ≥18 years and residence within the United States. Age and residence were assessed using screening questions without indication of eligibility criteria before commencement of the earliest survey (recontacted respondents: April 2–8, 2020; first-time respondents: June 24–30, 2020). Residence was reassessed among recontacted respondents during June 24–30, and one respondent whose primary residence had changed to outside of the United States was excluded from the analysis. Country-specific geolocation verification via IP address mapping was used to ensure respondents were from the United States. Informed consent was obtained electronically during June 24–30, 2020, before enrollment into the study as a participant. All surveys underwent Qualtrics, LLC data quality screening procedures including algorithmic and keystroke analysis for attention patterns, click-through behavior, duplicate responses, machine responses, and inattentiveness. Respondents who failed an attention or speed check, along with any responses identified that failed data quality screening procedures, were excluded from the analysis (6.6%).

** The COVID-19 Outbreak Public Evaluation (COPE) Initiative (https://www.thecopeinitiative.org/) is designed to assess public attitudes, behaviors, and beliefs related to COVID-19 pandemic and to evaluate the mental and physical health consequences of the pandemic. The COPE Initiative surveys included in this analysis were administered by Qualtrics, LLC (https://www.qualtrics.com), a commercial survey company with a network of participant pools comprising hundreds of suppliers and with varying recruitment methodologies that include digital advertisements and promotions, word-of-mouth and membership referrals, social networks, television and radio advertisements, and offline mail-based approaches. This analysis focused on questions about hand hygiene behavior during the COVID-19 pandemic.

†† 45 C.F.R. part 46, 21 C.F.R. part 56; 42 U.S.C. Sect. 241(d); 5 U.S.C. Sect. 552a; 44 U.S.C. Sect. 3501 et seq.

§§ https://www.medrxiv.org/content/10.1101/2020.04.22.20076141v1.

¶¶ https://www2.census.gov/geo/pdfs/maps-data/maps/reference/us_regdiv.pdf.

*** https://www.hrsa.gov/rural-health/about-us/definition/datafiles.html.

††† https://www.npr.org/sections/coronavirus-live-updates/2020/03/25/821513190/stop-price-gouging-33-attorneys-general-tell-amazon-walmart-others.

§§§ https://psyarxiv.com/dz428/.
